# Gendered (SDG5) and other perspectives on COVID-19 vaccination status: a focus on South Africa's Limpopo province

**DOI:** 10.3389/fgwh.2024.1420967

**Published:** 2024-07-30

**Authors:** Godwell Nhamo, Malebajoa Anicia Maoela

**Affiliations:** Institute for Corporate Citizenship, University of South Africa, Pretoria, South Africa

**Keywords:** COVID-19, SDG5, gender, stakeholders, age-oriented, pandemic, public policy

## Abstract

One of the key issues embedded in the 2030 Agenda for Sustainable Development is the need for disaggregated data. Given the nature of the Coronavirus disease 2019 (COVID-19), studies on such should respond to this call. This paper investigates gendered and other perspectives on COVID-19 vaccination status in South Africa's Limpopo Province. The work utilises a household survey (*n* = 4,571), data from Our World in Data and Johns Hopkins University, as well as policy documents and academic literature. The findings are that the government moved away from a goal to attain 67% herd immunity, to the containment strategy. While the country attained 35% of population fully vaccinated, the current study reveals 72.84% of the respondents fully vaccinated in Limpopo (including those receiving a booster). Noteworthy findings include 7.1% of the respondents reporting partial vaccination and 19.8% expressing vaccine hesitancy. Gender differences were significant, with females exhibiting higher vaccination rates than males, and age-related variations were observed, particularly among the youngest participants. Further analysis stratified by gender and age groups unveiled substantial disparities, emphasizing the need for targeted interventions. Additionally, the study highlights patterns in COVID-19 vaccine uptake based on education levels, with higher education associating with increased vaccination rates. Significant gender-based differences in vaccine uptake across education levels indicate potential areas for focused public health efforts. The findings emphasise the complexity of factors influencing vaccination behaviour, providing valuable insights for policymakers, public health practitioners, and researchers aiming to enhance vaccine uptake and address disparities in diverse demographic groups.

## 1 Introduction

The interfacing between gender and other perspectives on COVID-19 vaccination status remain of interest to key stakeholders, among such policy makers, academia, development agencies, and others. The interfacing is also important in the context of COVID-19 herd immunity, described as a point at which a community will no longer be vulnerable to threatening disease transmission, given the realisation of a certain vaccination threshold (1). Reinhardt and Rossmann (2) are clear that herd immunity through vaccinations had to be achieved if global leaders were to entertain getting back to pre-pandemic times.

The term *gender* appears 17 times in the 2030 Agenda for Sustainable Development, which enshrines 17 well-known, but difficult to separate Sustainable Development Goals (SDGs). The term appears to be coupled to concepts such as equality, gap, perspectives, empowerment, sensitive, disparities as well as age, race, ethnicity, migratory status, disability, geographical location and other relevant characteristics (3). Drawing even closer to the subject of investigation, SDG5 outlines the need to “achieve gender equality and empower all women and girls” [(3): 14]. In addition, linked to SDG5 and COVID-19 is SDG3, which looks at ensuring healthy lives and promoting well-being for all at all ages by 2030. Target 3.8 demands that the world attains universal health coverage. The target goes further, presenting a case for “access to quality essential health-care services and access to safe, effective, quality and affordable essential medicines and vaccines for all” (Ibid.: 14 & 16)—a matter that links directly with COVID-19 vaccinations.

Early work on gender and related perspectives surrounding COVID-19 vaccinations focused on the intention to get the dose. Acceptance to receive vaccines in the USA were generally high, with 81.1% of the responding 2,978 participants saying they would vaccinate (4). However, early studies exploring the determinants and hesitations surrounding COVID-19 vaccine acceptance across gender, age, and education levels uncovered significant insights and findings. Malik et al. (5) surveyed 672 adults in the USA and found that 67% of those responding would accept a COVID-19 vaccine if recommended for them by authorities. In addition, 72% males (compared to females) and 78% older adults (of 55+ years) compared to younger adults indicated they would take the vaccination. Lazarus et al. (6) found that women in France, Germany, Russia and Sweden indicated stronger willingness to accept COVID-19 vaccine than their male counterparts. Regarding age, the older age cohort (<50 vs. ≥50 years) remained a significant parameter in Canada, Poland, Sweden and the UK. However, in China, there was a reverse trend, with younger individuals more likely to accept a vaccine if available. Findings further revealed less significant differences for respondents aged <40 vs. ≥40. Mixed patterns have been observed across various education levels, indicating both positive and negative associations with COVID-19 vaccine uptake (7, 8).

From a sample of 2,368 respondents of 20 years and above, 53.1% were willing to take up vaccination in Kuwait (9). As for the gendered dimension, 58.3% of males (compared to 50.9% of females) were willing to take the vaccination. These findings reveal demographic disparities in vaccine acceptance. However, real data from the COVID-19 vaccines roll-out in the USA bring us closer to what took place on the ground. Diesel et al. (10) reflects on the work by the USA Centre for Disease Control (CDC), which analysed data of all adults of 18 years+ nationwide who were eligible for vaccination. It emerged that during 14 December 2020–22 May 2022, an estimated 57.0% of persons aged ≥18 years had received ≥1 COVID-19 vaccine dose. The spread was highest in the ≥65 years cohorts at 80.0%, and lowest in the 18–29 years cohort at 38.3%.

There has also been work done on sex/gender-disaggregated data following global calls for such by the United Nations (3, 11). Progress in this regard led to uncovering of important findings about COVID-19 testing, incidence, severity, hospitalisations and deaths. The current analysis reveals a scarcity of research on gendered and other perspectives related to the actual COVID-19 vaccination status. This gap is also extended to South Africa where the government remained opaque on matters gender, race, age, education levels, etc. Given the foregoing, this work seeks to examine gendered and other perspectives on COVID-19 vaccination status in South Africa's Limpopo Province. The work moves us from the national “COVID-19 backbox” to sub-national structures and the household.

In light of the preceding information, the following two objectives are spelt out: (1) To establish overall vaccination rates, and those between males and females respondents sampled from predominantly rural, vs., predominantly urban geographical set-ups; and (2) To analyse vaccination patterns across selected age groups and education levels that were part of the household survey respondents. The findings from the work have future policy implications, especially fulfilling some of the requirements towards the attainment of SDGs 3–5.

## 2 Literature review

This section reviews the literature and teases out perspectives on herd immunity, gender, education levels, age-oriented vaccination and other relevant factors (12). However, given the special attention group of pregnant women and breastfeeding mothers, space is also allocated for this, as such statistics form part of the entire vaccination spread. Paramita et al. (13) sum up the debate as follows: “The discourse of gender amidst the COVID-19 pandemic had been a big fuss. Amongst the discussions is the gender-related responses to COVID-19 that generally assume females to better respond to COVID-19 than males”.

### 2.1 The concept of COVID-19 herd immunity

One may not research gender and other COVID-19 vaccination status without touching on herd immunity. While the concept of COVID-19 herd immunity was applied largely at national level (14), it is logical that the same principle be applied at sub-national tiers of government. Herd immunity also comes with the baggage of vaccine efficacy and effectiveness (15), some perspectives that elevated the role and long road of regulatory clearances in many countries.

As for South Africa, the Bhekisisa Team (16) and Department of Health (17) identify 67% as the required COVID-19 herd immunity for the nation. This herd immunity was to be attained by December 2022. However, after realising that the target was illusive, the government changed the focus to containment (16). Containment would permit the health system to continue functioning without a total collapse, thereby managing the pandemic. The vaccination drive was derailed by several factors. For example, the first batch of the AstraZeneca vaccine was ineffective against South Africa's Beta variant, leading the department of health to sell the stock to other countries. The delayed J&J vaccine roll-out followed the need to investigate unusual blood clots by the South African Health Products Regulatory Authority (Sahpra). There was also vaccine nationalism, a phenomenon that witnessed the rich countries hoarding half of the global vaccines, despite these countries having less than 20% of the world's population (15). To rub salt into the wound, early online registration for vaccination was disturbed by the national load-shedding scheduling. This meant that officials had to update the system manually (16).

Providing an update in one of the national addresses (popularised as the COVID-19 Family Meetings) on 22 March 2022, the President acknowledged that more than 68% of people older than 60 years had been vaccinated (18). However, there was a concern regarding only 35% of people between 18 and 35 years having received a vaccination. Given the low uptake, the country launched the #KeReady campaign to encourage this age group to vaccinate.

Part of the challenges associated with the low vaccination uptake for the group under consideration could have been the vaccine roll-out strategy for the country. The age cohort under review was among the last to be enrolled. There were three vaccination phases designed to manage the limited COVID-19 vaccines stock. Phase 1 looked at vaccinating 1.25 million frontline workers (17). Phase 2 considered essential workers (2.5 million), those in congregate settings (1.1 million), those over 60 years (5 million), and those 18 years+ with comorbidities. Phase 3 included all adults, which were about 22 million.

### 2.2 Gender, education levels and age-oriented COVID-19 vaccination perspectives

Kaadan et al. (19) focused on the Arab world and how COVID-19 vaccine acceptance played out. The results from the 870 participants revealed vaccine acceptance at 62.4%, with males having a 65.4% acceptance rate. In addition, age group, level of education, and previous COVID-19 infection also stood out as factors influencing decision to take vaccines. In Israel, a national survey was done in October 2020 (20), using a sample of 957 adults aged 30 years+. From the sample, 606 respondents were Jews (49% males) and 351 were Arabs (38% males). Findings indicated that among men, 27.3% of the Jewish and 23.1% of the Arab respondents would opt for immediate vaccination, compared with only 13.6% of Jewish women and 12.0% of Arab women.

In France, Alleaume et al. (21) considered the general populace regarding investigating its intention to get vaccinated. A total of 5,018 participants were recruited in the survey, with 24% reporting their intention to refuse the vaccine. The main reason was concerns regarding the vaccine safety. In addition, women were more likely to refuse the vaccine. Work by Yoda and Katsuyama (22) narrowed down the focus to integrate gender and age group in vaccine acceptance in Japan. From a sample of 1,100 respondents, the authors found that 65.7% indicated a willingness to be vaccinated. Included in this group were older age groups, those in rural areas, and those with comorbidities.

In Malaysia, a total of 1,411 respondents of 18 years+ were sampled to take part in a similar survey (23). The distribution included young adults (40.7%), females (62.8%), Malay (63.8%), Muslim (72.3%), married people (52.9%), and those without medical illness (85%). It emerged that up to 83.3% of those sampled accepted vaccination, with 63.4% for the elderly (60 years+) cohort and 64.6% from the pensioners cohort. An estimated 24.7% and 23% of respondents who had comorbidities like diabetes mellitus and hypercholesterolemia, showed hesitancy of 16.1% and 15.8%, respectively. From a systematic review and Meta-Analysis study concerning COVID-19 vaccination acceptance from 172 studies drawn from 50 countries, Norhayati et al. (24) discovered overall acceptance of 61%. Additional findings were that the acceptance rate was higher in Southeast Asia, and for males this was so for vaccines with 95% effectiveness. In similar work applying the same methodology, Nehal et al. (25) sampled 411 articles that embedded 63 surveys from more than 30 countries. The global vaccination willingness stood at 66.01%. The authors also found that age, gender, education, attitudes and perceptions about vaccines were significantly associated with the acceptance or refusal rates.

Using vaccination data from the Maharashtra State of India, Potdar et al. (26) studied gender disparities towards the COVID-19 vaccination drive. The results showed that there were 84 women vaccinated for every 100 men; a ratio lower than India's gender ratio of 90:100. Foy et al. (27) looked at gender, age and COVID-19 vaccination status in African American Adult Faith-Based Congregants in the Southeastern USA. About 1,240 (70.9% women) adult congregants aged 18 years and older were sampled. Up to 86% of the respondents had received ≥1 dose of a COVID-19 vaccine. Higher odds of COVID-19 vaccination were aligned to increased age of women, while that association was not significant for man. While older adults are usually associated with higher rates of vaccination and/or wishing to be vaccinated, Fuller et al. (28) found that cost and needle phobia remain the most prevalent barriers from 901 older adults (aged 65+) surveyed in the state of North Dakota in USA. This could be possibly the reason why oral tablets have emerged as a form of COVID-19 vaccinations (29).

As regards the African continent, Tlale et al. (30) looked at the acceptance rate and risk perception towards the COVID-19 vaccine in Botswana. Up to 5,300 adults (3,199 females) were surveyed. Included in the group were 61% aged 24–54. The general vaccine acceptance rate was 73.4%, with men being more inclined to accept vaccination. Those aged 55–64 had high odds of accepting the vaccination when pitched against those aged 65 and above. Staying on the continent, 6 months into the rolling out of COVID-19 vaccination in Cameroon, “only 1.1% of the target population was fully vaccinated, with women representing less than one-third of the vaccinated population regardless of age, profession or comorbidities” (31). Women mentioned doubts about the quality or safety of the vaccine, the perception that they were being forced to vaccinate, and the variety of vaccines on the market, and belief that there were “more local” effective alternatives to the available vaccines.

Education levels play a pivotal role, correlating higher education with greater health literacy and preventive health measures, while lower education may contribute to vaccine hesitancy. The interplay of education and gender further complicates vaccination dynamics, necessitating tailored public health strategies. From a systematic literature review by Adu et al. (32), 18 studies found that education levels correlated positively with COVID-19 vaccine uptake, whereas three studies observed a negative relationship with education level.

### 2.3 Perceptions from pregnant, reproductive-age and breastfeeding women

A special group of people required a special consideration regarding COVID-19 vaccines uptake; thus, pregnant, reproductive-age and breastfeeding women. This group remains of interest because when statistics on vaccinations are presented as aggregated by gender, this group is is also included and accounts for a large proportion of the general population (33). Ayhan et al. (34) surveyed 300 pregnant women, with 37% revealing they would take up the vaccination if available. The main reason for the hesitancy among such group was vaccine safety.

Sutton et al. (33) surveyed 1,012 people in the USA, and out of this cluster, 46.9% identified as non-Hispanic White, 10.9% as non-Hispanic Black, 28.8% as Hispanic, and 8.2% as non-Hispanic Asian. An estimated 64.8% of the respondents were not pregnant, while 21.3% were pregnant, with the remaining 12.1% breastfeeding. It emerged that non-pregnant respondents were most likely to accept vaccination, and these took up 76.2% of this category. Those that were breastfeeding were the second most likely to vaccinate, with 55.2% of this group indicating so. On the other hand, pregnant respondents represented the lowest rate of vaccine acceptance, at 44.3% in that category.

A study in 16 countries^1^ brings interesting insights on the subject matter. In a November 2020 study, Skjefte et al. (35) surveyed 17,871 respondents. The findings revealed that at 90% COVID-19 vaccine efficacy, 52% of pregnant women (*n *= 2,747/5,282) and 73.4% of those non-pregnant (*n* = 9,214/12,562), respectively, would take the vaccine. Vaccine acceptance was generally highest in India, the Philippines, and all sampled countries in Latin America. However, it remained lowest in Russia, the USA and Australia. While vaccine acceptance rates vary among women globally, the confidence in vaccine safety and/or effectiveness remains among the strongest predictors of vaccine acceptance.

A PRISMA study, identifying 25,147 participating pregnant women from 17 out of 375 studies was conducted by Bhattacharya et al. (36). The sample studies covered four continents and the key finding was that only 49% of the women accepted the vaccination. Vaccination acceptance was also considerably low across specific sub-groups in the sample. Halemani et al. (37) conducted a similar PRISMA study that retrieved 26,995 articles, with 24 articles making it into the final sample. The 24 articles comprised a sample of 22,947 pregnant and 11,022 breastfeeding women. About 54% of the pregnant women and 59% of breastfeeding mothers, respectively, had the intension to take the COVID vaccination. Furthermore, from the pregnant mothers, 21% with comorbidities were also willing to take the COVID vaccine.

Work from Thailand showed an interesting pairing in a survey of pregnant women and intention to vaccinate. A total of 171 women and their 176 male partners were included; this after the exclusion of five pregnant women who had received a COVID-19 vaccination already (38). Up to 60.8% and 61.4% of the sampled women and their male partners, respectively, indicated they would vaccinate. Confidence in vaccine safety remained an associated factor affecting the husband's willingness to have his wife vaccinated during pregnancy. What was of interest, however, was that the actual rate of vaccination during pregnancy stood at a high of 88.3% in Thailand.

In summary, the risks of infections and comorbidities were some of the pull factors for accepting COVID-19 vaccinations in pregnant women. Likewise, and on the contrary, the adverse effects and safety concerns for COVID-19 vaccines were among the top indicators for the rejection of the COVID vaccine. Generally speaking, women are less likely to vaccinate compared to their male counterparts. Furthermore, older individuals are also likely to vaccinate than younger populations.

This literature section has been undertaken in order to interface it with the findings coming out of this work. The review further assisted in the discussions of the findings and strengthening of arguments.

## 3 Materials and methods

**Figure 1:**
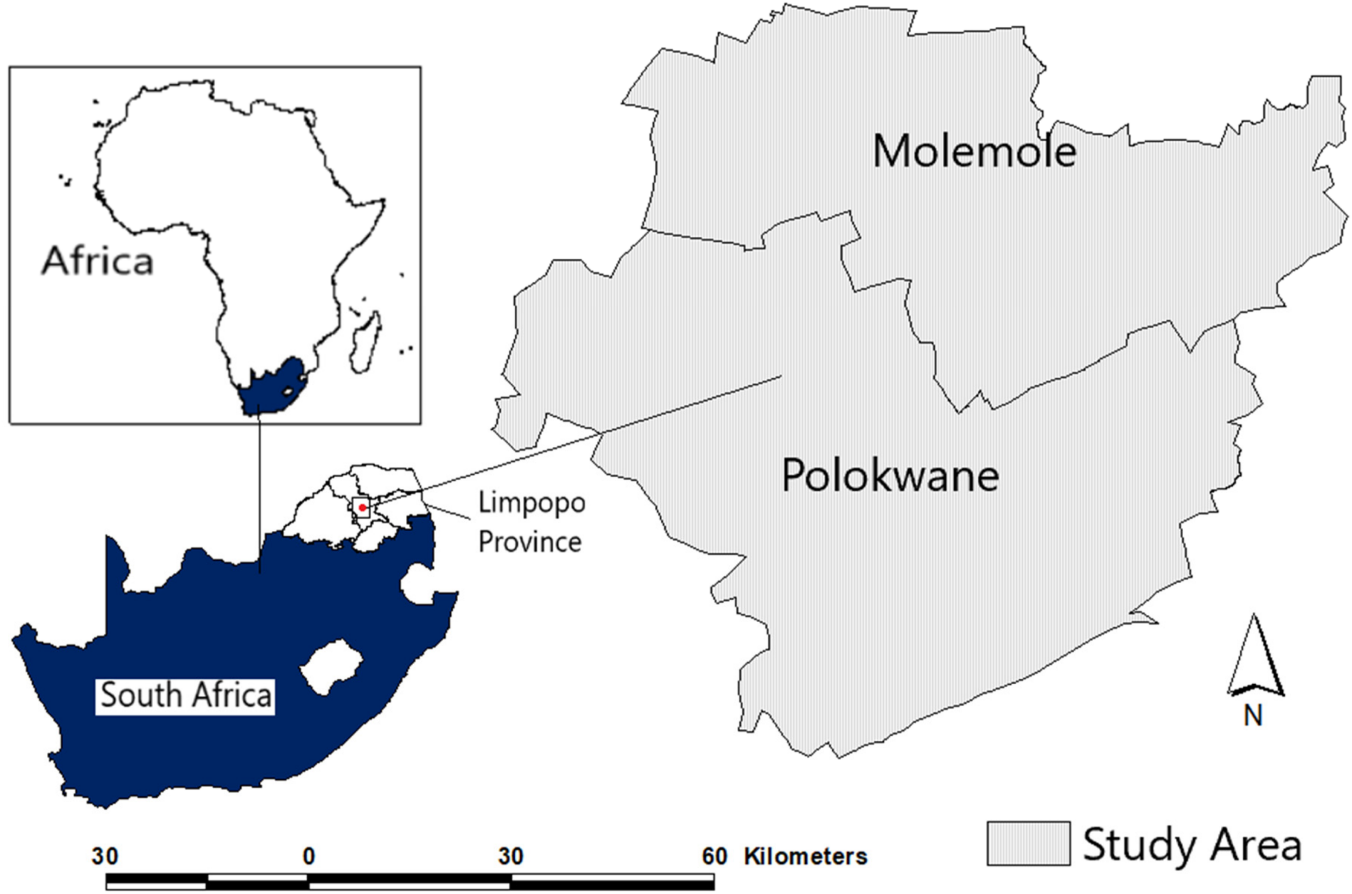
Location of the study area.

The entry point to the methodological orientation was understanding and embracing the call by the United Nations under the 2030 Agenda for Sustainable Development “to increase, significantly the availability of high-quality, timely and reliable data disaggregated by income, gender, age, race, ethnicity, migratory status, disability, geographic location and other characteristics relevant in national contexts” [(3): 27]. As authors, we believed that generating data concerning vaccination status in the selected Limpopo province's geographical locations in the form of Molemole (predominantly rural) and Polokwane (predominantly urban) Local Municipalities, was not only timely, but also needed to tease out gender and other facets. This way, the findings would be of value to the government and those reporting on the movement towards the attainment of the SDGs, especially SDG3, SDG 4 and SDG5. The broader study location is shown in Figure 1.

As advised by Paramita et al. (13), our conceptualisation of gender goes beyond the binary biological sex taking into consideration the multidimensional perspectives. While a question was pitched to generate data on sex, the respondents were given an option not to disclose their gender should they wished to do so.

To start the research, an ethics clearance was sought and granted by the Limpopo Government Research Ethics Committee on 20 July 2022 for Research Project No. LPREC/35/2022. A mixed-methods approach was adopted. The household survey was conducted between September and December 2022, during which time South Africa was in the process of rolling out its Covid-19 vaccination program, and the country was navigating various waves of infection. The sample included 4,571 households sampled using census approach following closely Statistics South Africa protocols and clusters. Inclusion criteria for survey participants meant respondents had to be a resident of the selected local municipalities and being aged 18 or older. The administered questionnaire covered a wide range of topics, including demographic information, vaccination status, sources of information about COVID-19 and barriers to accessing vaccination.

Descriptive summary statistics (counts and percentages) were computed to characterise the sample. For variables with measurable mean values, a one-way analysis of variance (ANOVA) was performed. Where the observed difference was significant, ANOVA was succeeded by *post-hoc* Tukey HSD pairwise *t*-tests, and the resulting *p*-values were adjusted for multiple comparisons. All analyses were performed using JMP Pro SAS for Windows, version 16.2.0.

In addition to the household survey, data from Our World in Data and Johns Hopkins University were used, alongside policy documents and academic literature. Among the policy proclamations considered were South Africa's President's regular updates on COVID-19 vaccinations, infections, deaths and recoveries. These proclamations were done through live televised and widely publicised so-called Family Gathering meetings.

## 4 Presentation of findings

### 4.1 The demographics

Since this work focused on gender and other parameters of interest, it was appropriate that respondents indicated their gender. Out of the 4,571 participants, 66.3% identified as females, 34% as males, and a minimal 0.1% expressed a preference not to disclose their gender. The data was further segmented by age cohorts, revealing that most respondents (19.5%) fell within the 18–29 age group, followed closely by the 30–39 age cohort at 18%. The distribution across age cohorts was generally well-balanced, ranging from 13.9% to 19.5%, excluding the outliers who opted not to disclose their age. Regarding education levels, secondary education was the most prevalent (41%), followed by primary education (28%). A detailed presentation of these findings is available in Table 1.

**Table 1:** Participant demographic information (*n* = 4,571).

Demographic variable	Frequency (% of valid)			Missing (% total)
	Urban	Rural	Full sample	
Gender				
Male	881 (36.1%)	655 (31%)	1,536 (34%)	11 (0.24%)
Female	1,558 (63.8%)	1,463 (69%)	3,021 (66.3%)	
Other	3 (0.1%)	0	3 (0.1%)	
Age (years)				
18–29	513 (21%)	376 (17.7%)	889 (19.5%)	6 (0.13%)
30–39	476 (19.5%)	340 (16%)	816 (18%)	
40–49	386 (16%)	311 (14.7%)	700 (15%)	
50–59	375 (15.3%)	353 (16.6%)	728 (16%)	
60–69	409 (16.7%)	371 (17.5%)	780 (17.1%)	
70+	276 (11.3%)	360 (17%)	636 (14%)	
Wish not to disclose	5 (0.2%)	11 (0.5%)	16 (0.4%)	
Level of education				
No formal education	287 (12%)	378 (18%)	665 (14%)	7 (0.15%)
Completed primary education	616 (25%)	660 (31.1%)	1,276 (28%)	
Completed secondary school education	1,035 (42%)	831 (39%)	1,866 (41%)	
Tertiary education	487 (20%)	243 (11.5%)	730 (16%)	
Other	19 (1%)	8 (0.4%)	27 (1%)	
Total	2,442 (54%)	2,118 (46%)	4,560 (100%)	24 (0.53%)

### 4.2 COVID-19 vaccination uptake: an overview

**Figure 2:**
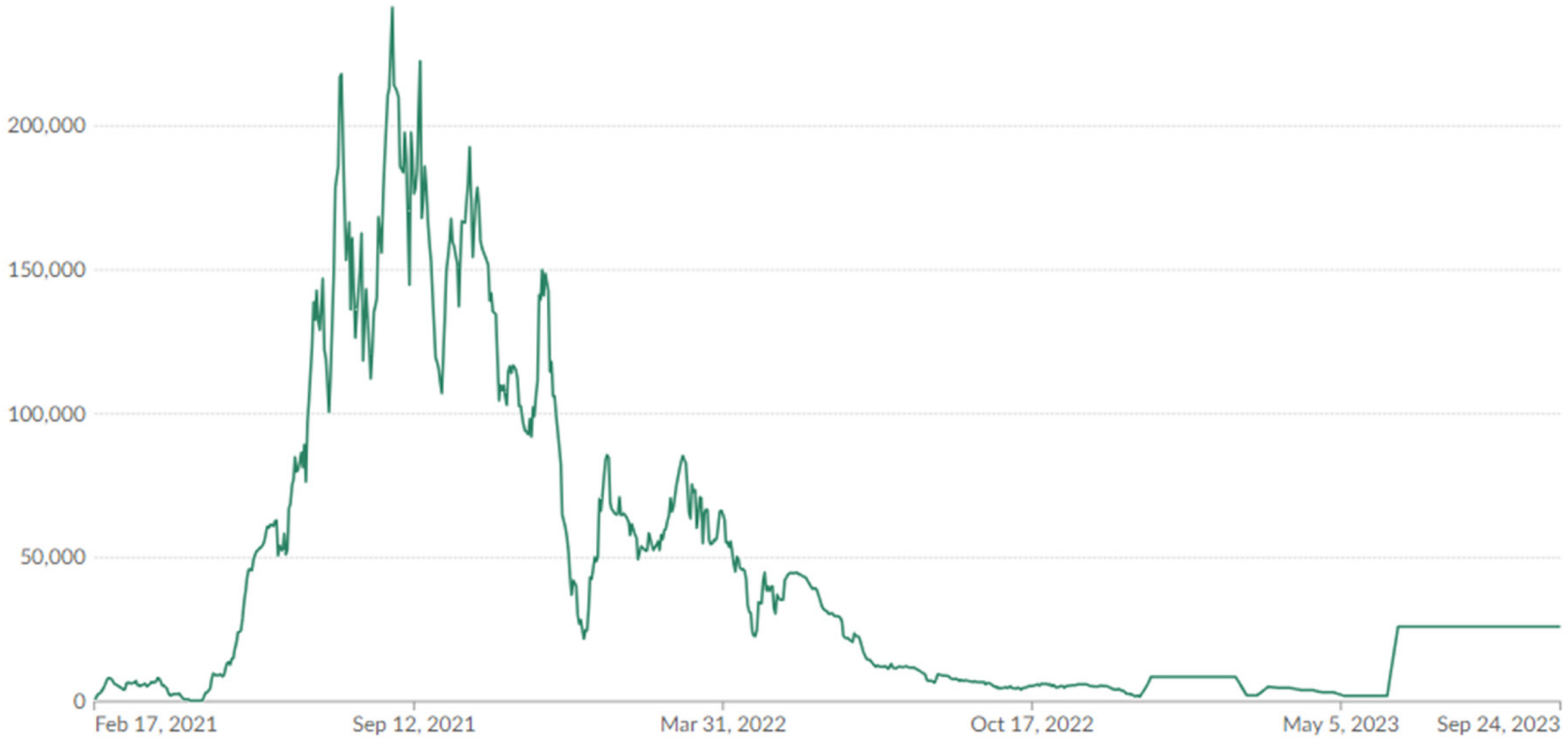
Daily COVID-19 vaccine doses administered in South Africa (2021–2023). Source: our World in Data (39), (https://ourworldindata.org/).

The general trend in COVID-19 vaccination status in South Africa is shown in Figure 2. The reported data covers all doses, including boosters, and was counted individually. The data is also on 7-day rolling averages. What is of further interest is that vaccinations in the country peaked in August 2021. As of 24 September 2024, the percentage of people with full vaccination stood at 35%, while those who were partially vaccinated were at 5.3%. This means that in total, 40.3% of the South African population were either fully and/or partially vaccinated. The percentage of the population receiving at least one dose corelates to that provided by Johns Hopkins University (40), which stood at 40.42% as of 3 October 2023, when the institution stopped collecting data. The coverage is based on about 38.58 million doses administered in the country. Furthermore, South Africa had reported 4.07 million cases of COVID-19 and 102,595 deaths.

**Figure 3:**
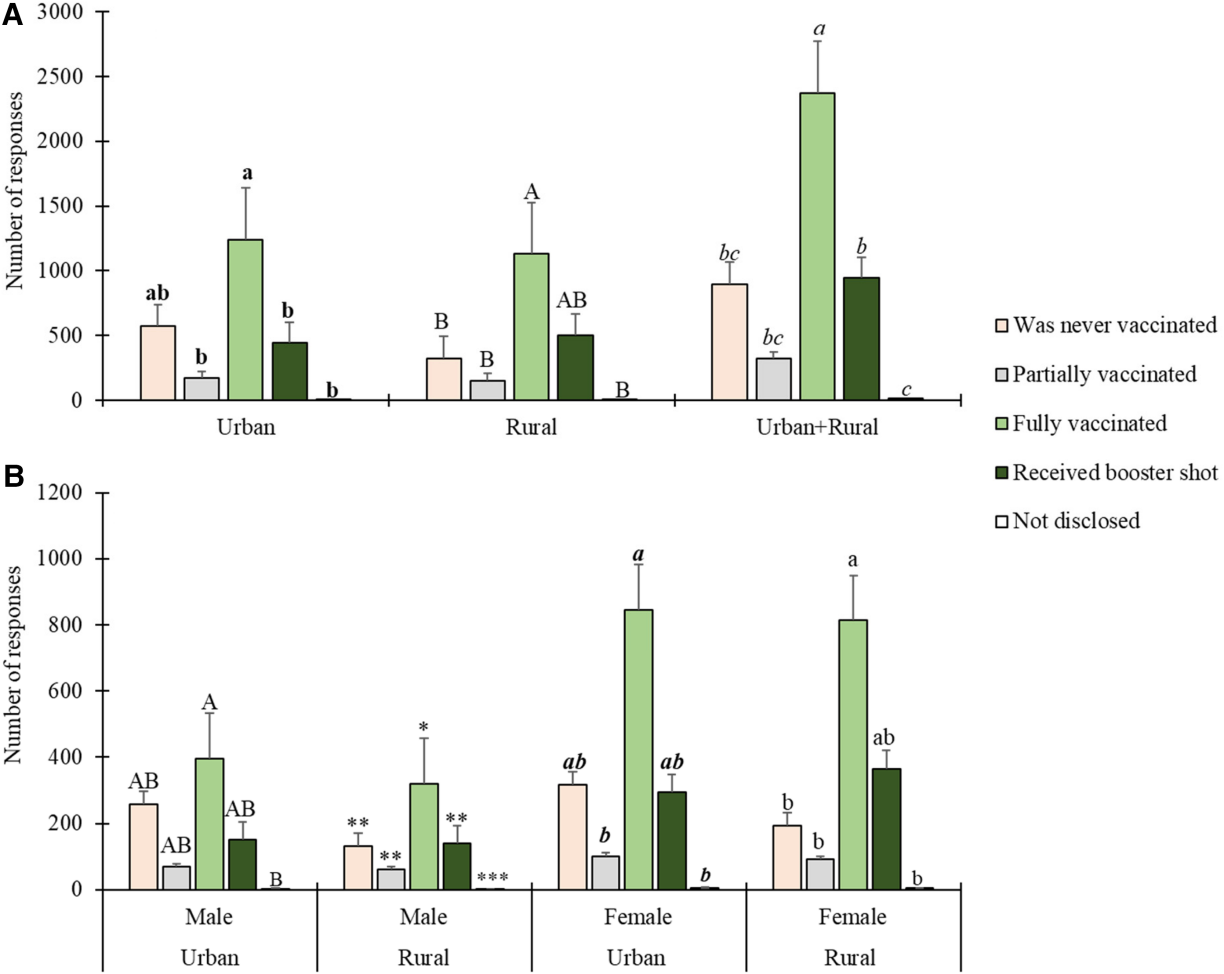
Respondents’ COVID-19 vaccination status by geographic location and gender. Statistically significant differences are indicated above error bars. Levels not connected by the same letter or number of asterisks are significantly different (*p* < 0.05). For vaccination status by geographic location in (**A**), differences in urban areas are indicated by bold lower-case letters, in rural areas by uppercase letters, and for urban and rural areas combined by italicised lower-case letters. For vaccination status by gender in (**B**), differences in male urban areas are indicated by uppercase letters, in male rural areas by asterisks, in female urban areas by italicised bold lower-case letters, and in female rural areas by normal lowercase letters.

Analysing the survey data, respondents were asked to indicate their vaccination status by choosing from five available options. The data was consolidated from respondents in two distinct municipalities, Molemole (rural) and Polokwane (urban). Despite no statistically significant difference between the two locations (*t* = 0.219, *p* = 0.832), the findings revealed that 52.1% of respondents were fully vaccinated, and an additional 20.8% had received a booster shot^2^. A more detailed breakdown is provided in Figure 3A, and the data is further categorized by gender in Figure 3B. Among the participants, 7.1% reported having received only partial vaccination, indicating incomplete immunization. Notably, the study identified vaccine hesitancy within the population, as 19.8% of respondents stated they had never been vaccinated. Additionally, a small percentage (0.4%) chose not to disclose their vaccination status.

### 4.3 Vaccination uptake by gender and their age cohorts

**Figure 4:**
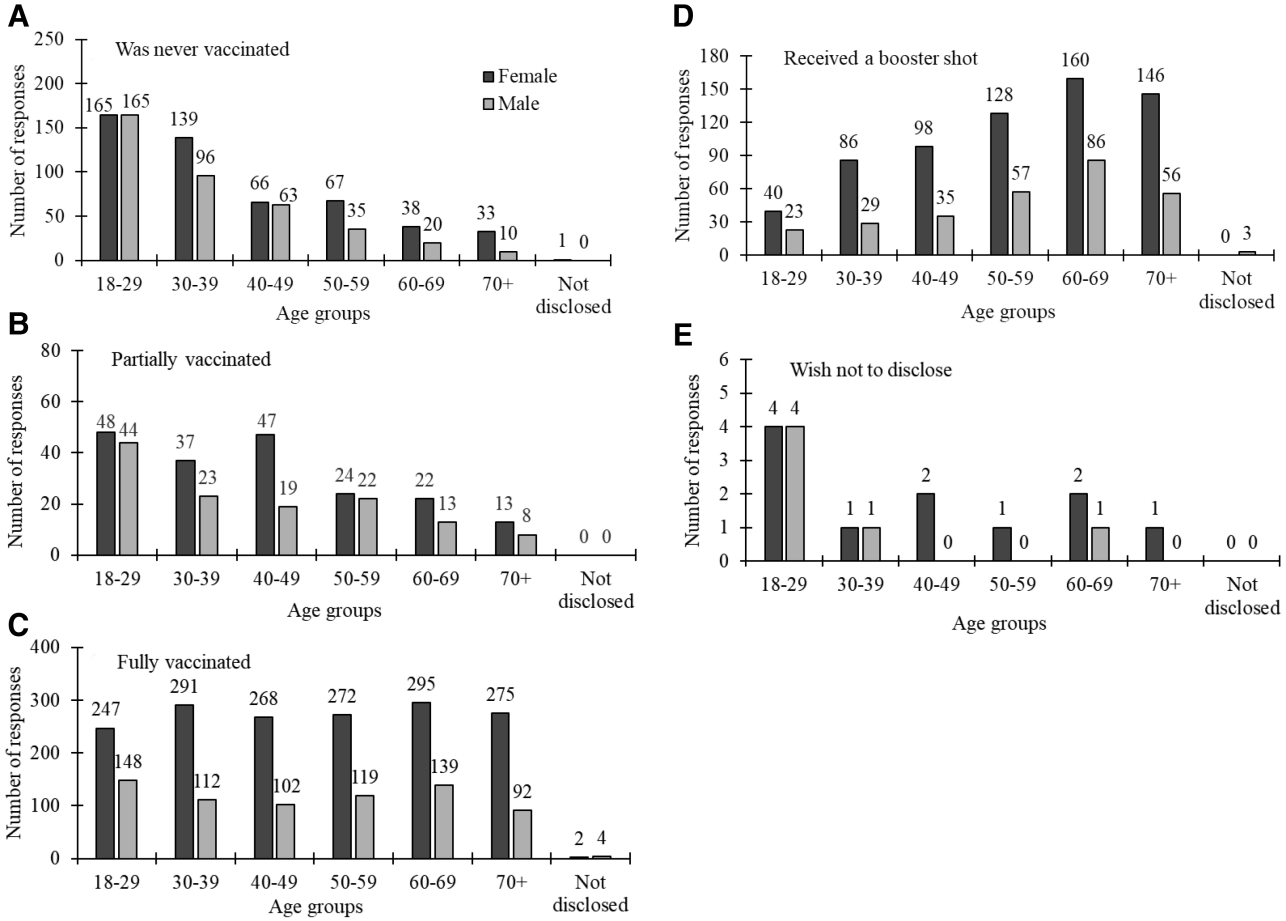
COVID-19 vaccination status by gender (dark grey bars for females and light grey for males) across different age groups.

Figure 4 presents descriptive statistics detailing the vaccination status among females and males, segmented by age groups. Notably, a substantial majority of participants in the youngest age bracket (18–39 years) reported never having been vaccinated, a trend observed in both females (*n* = 304) and males (*n* = 261). Conversely, individuals in the older to oldest age categories, particularly females aged over 40 years, exhibited a higher likelihood of having completed their vaccination series and having received a booster shot.

A one-way ANOVA stratified by gender for COVID-19 vaccination status yielded insightful results (Table 2). Significant associations between vaccination status and age groups were observed for males. Notably, a substantial gender-based disparity emerged among those who reported never being vaccinated and being partially vaccinated (*p* < 0.0001 for both), with *post-hoc* results indicating distinctions across various age groups. Similarly, fully vaccinated individuals (*p* = 0.037) and those who received a booster shot (*p* = 0.004) showed a gender-based difference, with specific age group comparisons shaping this variation.

**Table 2:** ANOVA results—summary of mean difference of vaccination status by gender across different age groups.

Vaccination status	Gender	Sum of squares	Mean square	*F*	Sig.	Tukey HSD *post-hoc* test results^a^
All	Male	7,853.6	1,308.9	2.27	0.048*	AG1–AG7
	Female	21,351.9	3,558.7	1.46	0.206	–
	All	25,503.9	4,250.7	2.69	0.017*	AG7–AG1, 2, 5
Was never vaccinated	Male	10,258.9	1,709.8	5.03	0.026*	AG1–AG6, 7
	Female	10,456.7	1,742.8	7.69	0.008*	AG7–AG1, 2; AG1–AG5, 6
	All	20,295.9	3,382.6	14.49	<.0001*	AG1–AG3, 4, 5, 6, 7; AG2–AG5, 6, 7
Partially vaccinated	Male	582.9	97.1	11.43	0.003*	AG1–AG3, 5, 6, 7
	Female	949.7	158.3	25.47	0.0002*	AG1–AG4, 5, 6, 7; AG2–7, 6; AG3–5, 4, 6, 7; AG4–AG7; AG5–AG7
	All	1,393.4	232.2	12.85	<.0001*	AG1–AG4, 5, 6, 7; AG2–AG7; AG3–AG6, 7; AG4–AG7
Fully vaccinated	Male	6,788.7	1,131.5	14.97	0.001*	AG7–1, 2, 3, 4, 5, 6
	Female	31,224.9	52.4.1	19.21	0.0005*	AG7–1, 2, 3, 4, 5, 6
	All	31,908.7	5,318.1	2.79	0.037*	AG5–AG7
Received booster shot	Male	2,226.7	371.1	23.51	0.0003*	AG7–3, 4, 5, 6; AG4–1, 7; AG5–1, 2, 4
	Female	10,024	1,670.7	7.9	0.008*	AG1–6; AG7–4, 6, 7
	All	10,580.4	1,763.4	4.56	0.004*	AG7–5, 6, 4; AG1–6
Not disclosed	Male	6.4	1.1	0.83	0.58	–
	Female	4.9	0.8	1.62	0.271	–
	All	10.4	1.7	2.56	0.051	–

^a^ Age groups: AG1 = 18–29, AG2 = 30–39, AG3 = 40–49, AG4 = 50–59, AG5 = 60–69, AG6 = 70+, AG7 = Not disclosed.

* *p* < 0.05.

Overall significant differences (*p* < 0.05) in vaccination status were observed between the youngest (AG1) and oldest (AG7) age groups for males, with no significant (*p* > 0.05) age group differences for females. When considering all subjects, significant differences were noted between the oldest age group (AG7) and several other age groups (AG1, AG2, AG5). For those who were never vaccinated, younger males (AG1) were less likely to be vaccinated compared to older males (AG6, AG7), and younger females (AG1, AG2) were less likely to be vaccinated compared to older females (AG5, AG6, AG7). Significant differences across multiple age groups were observed for both males and females. In partially vaccinated subjects, younger males (AG1) were less likely to be vaccinated compared to older males (AG3, AG5, AG6, AG7), and females showed significant variation in partial vaccination status across a wide range of age groups. Fully vaccinated males and females showed significant differences between the oldest age group (AG7) and all other age groups, indicating that older individuals were more likely to be fully vaccinated. When considering all subjects, significant differences were noted between age group 5 (AG5) and age group 7 (AG7). For those who received a booster shot, significant differences were observed among various age groups for both males and females, with broad variability in booster shot status across different age groups. In contrast, cases where vaccination status was not disclosed showed no statistically significant gender difference (*p* = 0.051), acknowledging associated limitations. Similar significant gender-based differences in vaccination status were observed across various categories.

### 4.4 Vaccination uptake by gender and levels of education

**Figure 5:**
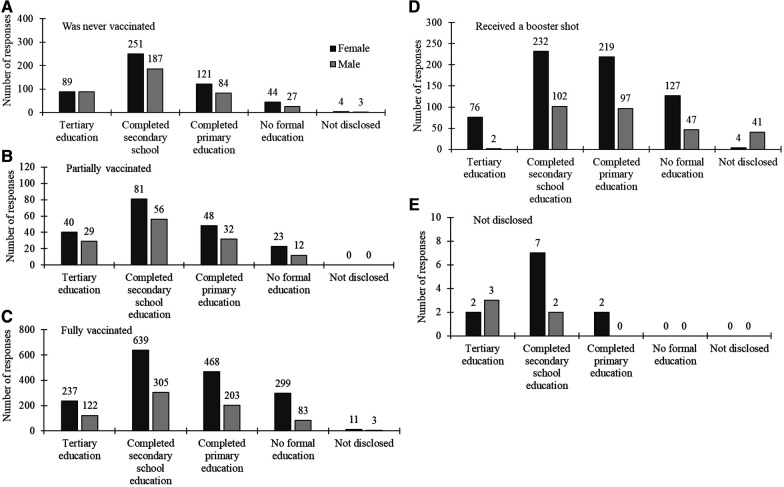
COVID-19 vaccination status by gender (dark grey bars for females and light grey for males) across different levels of education labelled in the x-axis.

The results related to COVID-19 vaccine uptake by gender and level of education revealed significant patterns. Notably, both males and females with higher education levels had the highest full vaccination count, and a similar trend was observed for those who indicated to have never been vaccinated. The reduced number of males compared to females within higher education levels indicated tthat they have not received a booster shot. A few males chose not to disclose their status, which poses an intriguing area for further investigation. The results are illustrated in Figure 5.

Table 3 complements the findings in Figure 5 by summarising the statistical results for the association between vaccination status, gender and levels of education, including significant *post-hoc* test results denoting specific level of education comparisons. The ANOVA results reveal significant differences in mean scores based on overall vaccination status among participants (*p* < 0.0001). *Post-hoc* tests highlight specific gender disparities, with noteworthy differences between education levels, such as Q3 to Q1 and Q2 to Q5. For those never vaccinated, fully vaccinated and those who received a booster shot, gender comparisons showed statistically significant differences with *p*-values close to conventional significance thresholds (*p* < 0.0001, *p* = 0.002 and *p* = 0.005 respectively), emphasising variations across education levels. In cases where vaccination status was undisclosed, a marginally significant gender difference was observed (*p* = 0.04), though specific pairwise differences were not identified in *post-hoc* tests. Similar significant gender-based differences in vaccination status were observed across various categories.

**Table 3:** ANOVA results: summary of mean difference of vaccination status by gender across different levels of education.

Vaccination status	Gender	Sum of squares	Mean square	*F*	Sig.	Tukey HSD *post-hoc* test results^a^
All	Male	23,991.7	5,997.9	5.23	0.002*	Q3–Q1, 5
	Female	81,189.5	20,297.4	3.75	0.01*	Q3–Q5
	All	95,674.1	23,918.5	6.95	<.0001*	Q3–Q1, 4, 5; Q2–Q5
Was never vaccinated	Male	10,121.4	2,530.4	4.19	0.074	–
	Female	17,849.4	4,462.3	8.95	0.017*	Q3–Q1, 5
	All	27,251.8	6,812.9	14.7	<.0001*	Q3–Q1, 2, 4, 5; Q2–Q5
Partially vaccinated	Male	908.4	227.1	9.27	0.016*	Q3–Q1, 5
	Female	1,810.6	452.7	46.19	0.0004*	Q3–Q1, 2, 4, 5; Q2–Q5; Q4–Q5
	All	2,636.7	659.2	21.86	<.0001*	Q3–Q1, 2, 4, 5; Q2–Q5; Q4–Q5
Fully vaccinated	Male	26,742.4	6,685.6	30.56	0.001*	Q3–Q1, 4, 5; Q2–Q1, 5; Q4–Q5
	Female	1,12,946.4	28,236.6	25.29	0.002*	Q3–Q1, 4, 5; Q1–Q5; Q2–Q5
	All	1,23,249.5	30,812	6.89	0.002*	Q3–Q2, 5; Q2–Q5
Received booster shot	Male	3,501.4	875.4	12.97	0.008*	Q5–Q2, 3
	Female	18,556.6	4,639.2	12.67	0.008*	Q3–Q4, 5; Q2–Q5
	All	19,012.8	4,753.2	5.93	0.005*	Q5–Q2, 4
Not disclosed	Male	4	1	2	0.23	–
	Female	16.4	4.1	3.15	0.12	–
	All	14.7	3.68	3.34	0.04*	–

^a^ Levels of education: Q1 = no formal education, Q2 = completed primary education, Q3 = completed secondary school education, Q4 = tertiary education, Q5 = not disclosed.

* *p* < 0.05.

Zooming into differences across qualification levels as shown in Table 3, for the overall population, males show significant differences between qualifications Q3 and Q1, and Q3 and Q5, while females exhibit differences between Q3 and Q5. When considering the combined data for all genders, significant differences are noted across multiple qualification levels (Q3–Q1, Q3–Q4, Q3–Q5, Q2–Q5). For those who were never vaccinated, females show significant differences between Q3–Q1 and Q3–Q5, while males do not exhibit significant differences. Among partially vaccinated individuals, males have significant differences between Q3–Q1 and Q3–Q5, and females show differences across several qualification levels (Q3–Q1, Q3–Q2, Q3–Q4, Q3–Q5, Q2–Q5, Q4–Q5). For fully vaccinated individuals, both males and females exhibit significant differences across multiple qualification levels, with males showing differences between Q3–Q1, Q3–Q4, Q3–Q5, Q2–Q1, Q2–Q5, Q4–Q5, and females showing differences between Q3–Q1, Q3–Q4, Q3–Q5, Q1–Q5, Q2–Q5. Those who received a booster shot also show significant differences, with males differing between Q5–Q2 and Q5–Q3, and females between Q3–Q4, Q3–Q5, and Q2–Q5.

## 5 Discussions

Globally, authorities have been proactively addressing the challenges posed by the COVID-19 pandemic through active promotion of vaccination efforts. Understanding the dynamics of vaccine uptake is crucial for informed decision-making, resource allocation and targeted strategy formulation. Recognizing age, education level and gender as key determinants influencing vaccine acceptance, research emphasizes the significance of comprehending these factors for effective crisis navigation, optimized public health outcomes and global efforts to mitigate the pandemic's impact (41, 42). This study investigated the associations among age, education level and COVID-19 vaccination status in both urban and rural areas of Limpopo province, South Africa, with a specific focus on understanding the moderating role of gender in these dynamics.

The study yielded encouraging results, revealing that about 73% of the sampled population had received full COVID-19 vaccination (including those who had a booster). The percentage of individuals refusing the vaccine fell within the internationally reported range [e.g., (43, 44)]. These results provide valuable perspectives on the ongoing and complex discussion on the factors that impact COVID-19 vaccination, especially in the context of gender (27, 45). While most of the respondents were fully vaccinated and have received a booster shot, over a quarter were not. In addition to their individual risk from vaccine-preventable disease, this group reduces herd immunity**.** Future studies should look at the factors related to this resistance to COVID-19 vaccines. Numerous studies [e.g., (32)] and references therein) have already indicated a connection between vaccine hesitancy and factors such as misinformation, distrust in vaccine efficacy or safety, cultural beliefs and social influences. Understanding these underlying elements can inform targeted interventions and communication strategies aimed at addressing vaccine hesitancy and promoting broader vaccination coverage to safeguard public health.

This study contributes evidence on gender disparities in vaccine uptake, revealing a higher rate of full vaccination among females compared to males. There is a gender disparity evident in the relationship between age and vaccination status among males. Interestingly, contrary to our expectations, no such age-related association was found among females across the five vaccination statuses. These findings prompt further consideration of potential explanations for this outcome. As per Ishimaru et al. (46), women aged below 49 years of age are more inclined towards proactive preventive behaviours, but they exhibit lower willingness to get vaccinated compared to men (which is why we expected to see the differences between age groups). However, when examining differences within individual vaccination status across all the considered age groups, it becomes apparent that the majority of respondents aged 18–39 years were never vaccinated and are hesitant to disclose their vaccination status, compared to those aged 40 years and above. These findings align with studies conducted in the US, UK, and Ireland (5, 47, 48). For example, a study conducted in the United States in May 2020 discovered that individuals aged 55 and above were more receptive to receiving a COVID-19 vaccine, compared to those aged 18–54 (5). Likewise, the United Kingdom reported lower vaccine resistance among individuals over 65, whereas in Ireland, those aged between 25 and 44 showed higher resistance (47). These outcomes may suggest a clear and effective communication of the vulnerability of the elderly to COVID-19.

The lack of disparities in immunization status between urban and rural residents observed in this study contrasts with some existing literature that suggests differences in healthcare access and vaccine uptake between urban and rural populations (49, 50). This finding may reflect effective national vaccination efforts in South Africa, including robust outreach programs that ensured equitable vaccine access across different geographic settings (51). The consistent communication of COVID-19 vaccination benefits and risks could also have contributed to uniform uptake rates. Future research could explore these dynamics further to ascertain whether similar patterns persist across different regions and time periods.

Another significant finding of this study pertains to the gender gap in the associations between education levels and vaccination status. Both females and males with tertiary education as well as those with primary and secondary education demonstrated higher rates of full vaccination and booster uptake. However, the gap between genders in vaccination rates was more pronounced among those with lower levels of education. Females with primary or secondary education showed significantly higher vaccination rates compared to their male counterparts. This aligns with prior research suggesting that a higher level of education serves as a facilitator for health protection and is consequently associated with greater intent for COVID-19 vaccination (5, 52). These findings highlight the significance of educational attainment in influencing vaccination behaviour and contribute to the broader understanding of factors shaping COVID-19 vaccination intentions. The observed gender gap suggests that females, even at lower education levels, might be more receptive to health messaging and vaccination campaigns than males. This contributes to the broader understanding of factors shaping COVID-19 vaccination intentions and emphasises the need for tailored health interventions that consider both educational and gender differences to improve vaccine uptake.

The study's primary strength lies in its substantial number of participants. Moreover, it was conducted during a period when information about COVID-19 and its vaccines was relatively stable, providing a consistent backdrop for analysis. Additionally, the utilisation of physically administered surveys could have potentially enhanced the response rate. This study is not without its limitations. The respondents were predominantly female, with education levels limited to primary and secondary school, making them non-representative of the broader population. Potential response bias may exist, favouring those with a positive stance on vaccination. It is important to acknowledge that the sensitive nature of COVID-19 vaccination as a survey question might have influenced the responses.

## 6 Conclusions

This study provides crucial insights into the dynamics of COVID-19 vaccine uptake, particularly in the context of age, education level, and gender disparities in both urban and rural areas of Limpopo province, South Africa. The findings emphasise the importance of understanding these factors for informed decision-making, resource allocation and the development of targeted strategies to combat the ongoing pandemic. Policy implications of these findings are noteworthy. Firstly, recognizing the higher rate of full vaccination among females compared to males suggests the need for targeted vaccination outreach and education campaigns tailored to specific gender demographics. Understanding the age-related associations and the observed hesitancy among younger age groups emphasizes the importance of tailoring communication strategies to effectively convey the vulnerability of the younger population to COVID-19. Additionally, the association between educational attainment and vaccination status highlights the role of education as a facilitator for health protection. Policies promoting education and awareness campaigns can potentially enhance vaccine acceptance across diverse populations. Moreover, acknowledging the limitations of the study, including the non-representative sample and potential response bias, emphasizes the importance of employing diverse and inclusive survey methods to obtain a more accurate representation of the general population. To address resistance to COVID-19 vaccines, future research should delve deeper into the factors influencing hesitancy, considering misinformation, trust issues, cultural beliefs and social influences. This understanding can inform targeted interventions and communication strategies to address vaccine hesitancy and enhance broader vaccination coverage. In navigating the complexities of the ongoing crisis, the evidence presented in this study offers a valuable contribution to the global discourse on COVID-19 vaccination, guiding policymakers and public health officials in their efforts to curb the impact of the pandemic and to promote public health.

## Data Availability

The raw data supporting the conclusions of this article will be made available by the authors upon reasonable request.

## Footnotes

^1^USA, India, Brazil, Russia, Spain, Argentina, Colombia, UK, Mexico, Peru, South Africa, Italy, Chile, Australia, New Zealand and the Philippines.

^2^For the purposes of herd immunity, those receiving a booster short are also considered as being fully vaccinated.
